# Chemical methods for determining the electron storage capacity of black carbon

**DOI:** 10.1016/j.mex.2018.11.007

**Published:** 2018-11-17

**Authors:** Danhui Xin, Minghan Xian, Pei C. Chiu

**Affiliations:** a Department of Civil and Environmental Engineering, University of Delaware, Newark, DE, 19716, United States; b Department of Chemical and Biomolecular Engineering, University of Delaware, Newark, DE, 19716, United States

**Keywords:** Chemical method, Black carbon, Biochar, Electron storage capacity, Electron accepting capacity, Electron donating capacity, Redox reversibility, Chemical oxidation/reduction

## Abstract

Electron storage capacity (ESC) is a new and important property that determines the capacity of a black carbon to mediate abiotic and microbial electron transfer reactions in natural and engineered systems. It is necessary to develop accurate and reproducible methods to measure black carbon's ESC in order to understand its redox behavior and to predict its capacity to support redox transformation of contaminants in subsurface environments. In this study, we developed chemical methods that employed combinations of reductants and oxidants of different redox potentials – Ti(III) citrate or dithionite as reductant, and ferricyanide or dissolved O_2_ as oxidant – to measure the ESC of a wood-derived biochar. Pore diffusion within biochar particles was rate-limiting and controlled the timescale for redox equilibrium and ESC measurements.

• *The new methods can handle sample mass on the order of a gram*
• *Sample pretreatment (e.g., oxidation via aeration) is necessary to produce consistent results*
• *For a given reductant-oxidant pair, colorimetric (or potentiometric) measurements gave constant and reproducible ESC*

## Graphical abstract

**Figure:**
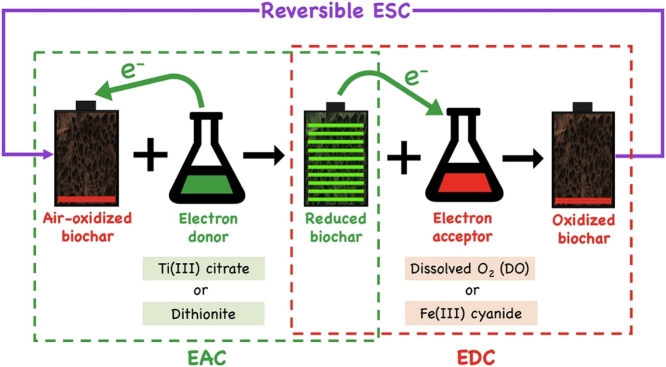

Specifications table

**Table:** 

**Subject Area**	• *Environmental Science*
**More specific subject area:**	*Environmental Chemistry*
**Method name:**	Chemical method
**Name and reference of original method**	Klüepfel L., Keiluweit M., Kleber M. and Sander M., Redox properties of plant biomass-derived black carbon (Biochar). *Environ. Sci. Technol.***48** (10), 2014, 5601-5611.
**Resource availability**	

## Method overview

Black carbon such as biochar can serve as an electron donor and/or acceptor in abiotic and microbial redox reactions. [[1], [2], [3]] Electron storage capacity (ESC) is an important property that determines a biochar's capacity to accept and donate electrons reversibly and support redox transformations [1,3,4]. It is important to develop accurate and robust methods to determine black carbon's ESC in order to evaluate the redox capacity of black carbon in sediment and soil and to correctly design biochar-based engineered systems for contaminant remediation. [[3], [4], [5], [6]]

Mediated electrochemical analysis (MEA) is the established method for measuring biochar's electron accepting capacity (EAC) and donating capacity (EDC). MEA is a sensitive technique that handles a small sample mass (<0.1 mg) [1]. For biochar produced in large quantities for field applications, the properties can be heterogeneous, and methods that can handle larger sample masses are needed to obtain a representative ESC.

In theory, ESC is the sum of EAC and EDC if the capacity is completely reversible. However, often only single EAC and EDC measurements were made for each biochar sample, and samples were used as prepared without pre-conditioning; i.e., without oxidation or reduction to bring samples to a pre-determined redox state prior to measurement [1,2]. Without bringing black carbon to a reference state before each analysis, any measured EAC/EDC would reflect the redox state and electron content of the sample *at the time of measurement* and would vary with storage, exposure to air and moisture, etc. In addition, the extent of reversibility of black carbon ESC has not been assessed, as few EAC and EDC measurements were conducted over multiple redox cycles on the *same* samples.

In this study, we employed pairs of chemical reductants (Ti(III) citrate or dithionite) and oxidants (ferricyanide or dissolved oxygen (DO)) of different redox potentials to assess the ESC of a wood biochar and its reversibility. These reductants and oxidants were selected because they (1) are sufficiently reducing [7,8] or oxidizing [9,10] relative to the reported reduction potentials of humic acids [11], (2) except DO, can be quantified by spectrophotometric and/or potential measurements, and (3) are anionic and thus would adsorb minimally to biochar. Soil Reef biochar (SRB), [3,12] prepared through pyrolysis of hardwood chips at 550 °C, was used in this method development/evaluation. SRB was drained of electrons through extended exposure to DO prior to experiments, and EAC and EDC measurements were repeated for the same SRB samples to evaluate the reversibility of its ESC. We believe the methods reported here can also be used to measure the ESC of other black carbons, including granular activated carbon (GAC).

### Method details

**Fig. 1:**
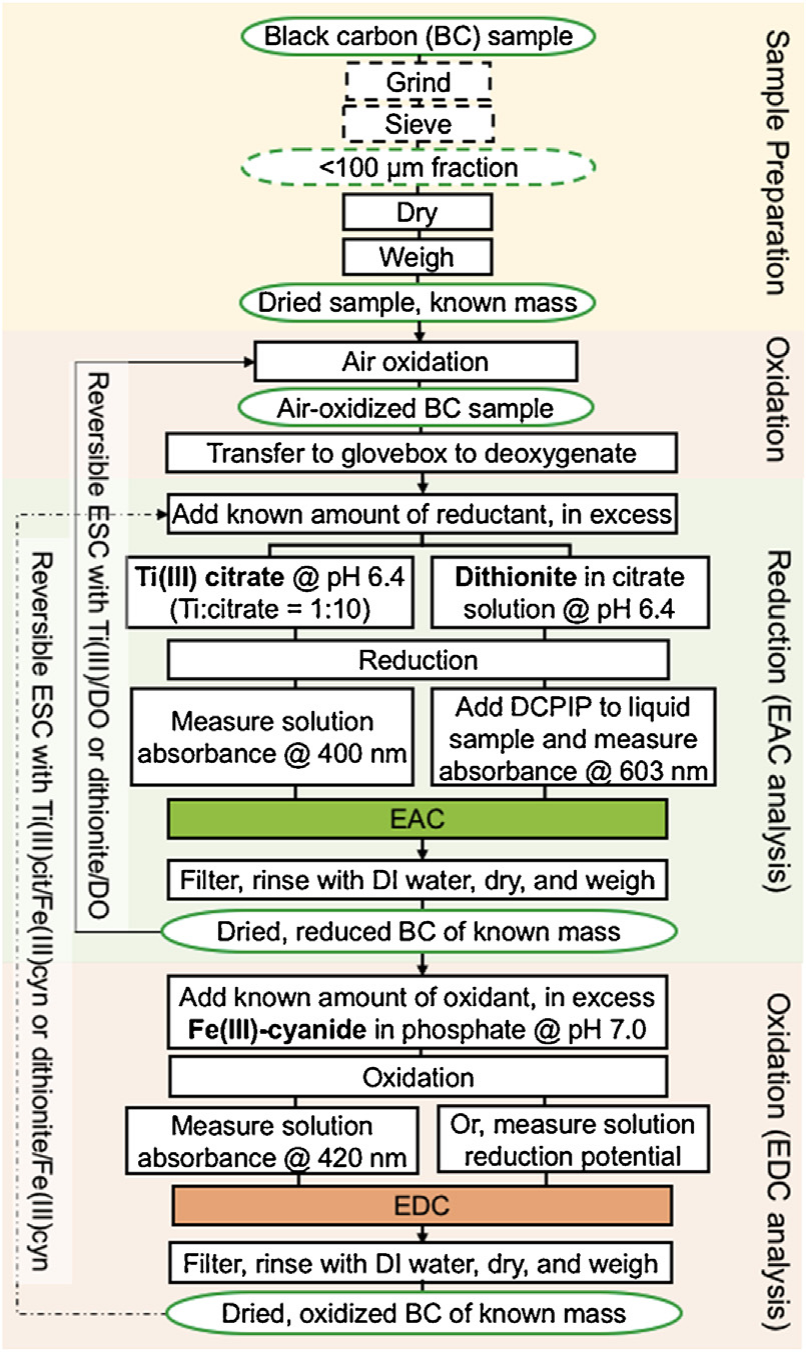
Experimental steps in ESC measurements.

The experimental steps involved in biochar ESC measurements are summarized in Fig. 1.

#### Chemicals

Titanium(III) chloride (20% w/v in 2 N HCl), titanium(IV) chloride (0.09 M in 20% HCl), sodium citrate (99%), potassium ferricyanide (99+%), 1,4-benzoquinone (99%), potassium ferrocyanide (98.5%), monobasic (98+%) and dibasic (≥99%) sodium phosphate, and sodium 2,6-dichlorophenol-indophenol (DCPIP, 98%) were acquired from ACROS Organics (Morris Plains, NJ). Sodium dithionite (>85%) was purchased from Alfa Aesar (Haverhill, MA). All chemicals were used as received.

#### Biochar preparation

SRB [3,12] was sieved and different size fractions were collected and dried at 65 °C for 24 h. The <100 μm size fraction was used in this investigation due to the shorter equilibration times. Dried SRB samples were stored in a desiccator, which resulted in a moisture content of 2.2 ± 1.5%, and weighed right before use. All reported EAC, EDC, and ESC values are based on dry weight.

#### Biochar oxidation with DO

SRB samples were oxidized by DO in a continuously aerated deionized (DI) water for 72 h, and then vacuum-filtered and dried (see above) before use. Note that the time needed for this pre-oxidation can vary from 1 to 5 days depending on biochar particle size and properties; e.g. pore size distribution and tortuosity. Long oxidation time would ensure all electrons in the sample are drained; i.e., all redox-facile functions in black carbon that are accessible to and oxidizable by DO are oxidized prior to measurement.

#### EAC and EDC measurements

All EAC and EDC were measured in an anaerobic glove box (2.0 ± 0.5% H_2_ in N_2_, P_O2_ <25 ppm, Coy, MI). Aqueous Ti(III) citrate and ferricyanide concentrations were quantified directly by UV–vis absorbance and/or reduction potential, whereas the *electron content* of dithionite solution was measured indirectly using DCPIP (described below). The wavelengths and extinction coefficients used to quantify 1,4-benzoquinone, Ti(III), DCPIP, and ferricyanide are shown in Table 1. Absorbance was measured using a Vernier LabQuest 2 UV–vis spectrophotometer (Vernier, OR), and reduction potentials were measured using an Orion ORP electrode (Thermo-Fisher, MA).

**Table 1:** Wavelengths and extinction coefficients of the oxidants and reductants used.

	Extinction coefficient (M^−1^ cm^−1^)^a^	Wavelength (nm)	R^2^
1,4-benzoquinone	32.4 ± 0.7	400 nm	0.999
Ti(III) citrate	91.5 ± 3.3	400 nm	0.996
DCPIP	18,600 ± 660	603 nm	0.996
[Fe(III)(CN)_6_]^3−^	1160 ± 27	420 nm	0.999

a Measured in this study and reported as mean ± one standard deviation. Calibration curves for the oxidants and reductants are given in Supplementary Data (Fig. S1–S4).

##### EAC measurement using Ti(III) citrate

Because we observed variations in Ti(III) concentration among different commercial sources and between batches of the same product, Ti(III) citrate concentration in each stock solution was first standardized by benzoquinone of high purity (Fig. S1, Supplementary Data). Each EAC measurement was performed in duplicates, and reactors without SRB were used as control for all experiments. Pre-oxidized SRB was added to 0.50 L of 10 mM Ti(III) in 100 mM sodium citrate at pH 6.4 ± 0.2 (the third pK_a_ of citric acid). Reactors were placed on an orbital shaker at 100 rpm. At different elapsed times, 6.6-mL samples were collected and passed through a 0.22-μm PVDF syringe filter for Ti(III) analysis. In addition to buffering solution pH and ionic strength, the excess citrate served to prevent sorption of Ti(III) citrate to SRB, which could be a source of error in EAC measurement. We have shown that Ti(III) citrate (and ferricyanide) did not sorb to SRB under our experimental conditions. [12]

The electrons transferred to SRB from Ti(III) were calculated based on electron balance using Eq. (1).

(1) $$e^{-}\;transferred\;(mmol)=C_{1}\times V_{1}-C_{n}\times \left(V_{1}-nV_{i}\right)-\sum_{i=1}^{n}\left(C_{i}\times V_{i}\right)$$

where C_1_ and V_1_ are the initial Ti(III) concentration and solution volume, respectively, n is the total number of samples, C_n_ is the final Ti(III) concentration at equilibrium, and C_i_ and V_i_ are the Ti(III) concentration and volume of the *i*th sample, respectively. That is, the amount of electrons accepted by SRB was taken to be the difference between the initial and final electron contents of the Ti(III) citrate solution minus the electrons contained in all the samples withdrawn, which collectively accounted for <10% of the initial electron content of the Ti(III) solution. SRB was taken to be at equilibrium with solution when the change in EAC calculated from Eq. (1) between two consecutive samples was <3%.

##### EAC measurement using dithionite/DCPIP

**Fig. 2:**
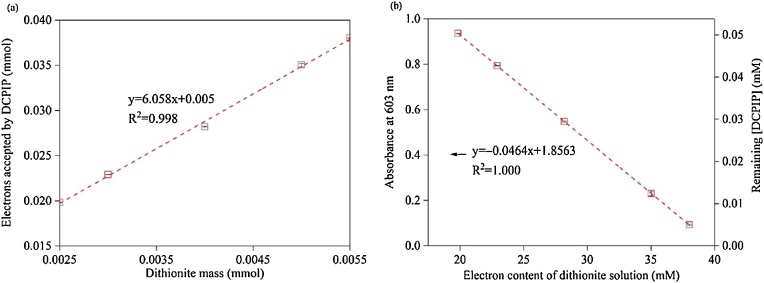
(a) Titration of dithionite samples using DCPIP. The slope shows that *ca*. 6 moles of electrons were transferred to DCPIP from each mole of dithionite, and that dithionite and its daughter reductants were fully oxidized to sulfate by DCPIP. (b) Calibration curve for the electron content of dithionite solution in 50 mM citrate buffer (pH 6.4) based on DCPIP absorbance at 603 nm. Error bars represent one standard deviation.

We also developed a method using DCPIP to quantify the *electron content of dithionite solution*, instead of measuring dithionite itself. This was necessary because (1) the purity of commercial dithionite is low (∼85%), (2) dithionite is unstable in solution and homolyzes to sulfoxyl radical (^•^SO_2_**^–^**) and disproportionates to thiosulfate and sulfite [13], and (3) unlike Ti(III) and Fe(III), which transfers one electron each, the number of electrons transferred per dithionite (where S redox state = +3) depends on the products formed; e.g., thiosulfate (S redox state = +2), sulfite (+4), or sulfate (+6). Dithionite and its daughter reductants can be oxidized fully by DCPIP (E_h_^o^ = +0.25 V at pH 7.0), [14] which can be quantified spectrophotometrically (Fig. 2). As SRB was reduced in dithionite solution, solution electron content was measured over time based on changes in DCPIP absorbance at 603 nm, and the EAC of SRB was calculated from the initial and final (i.e., equilibrium) solution electron contents.

Each EAC measurement was performed in triplicates, and reactors without SRB were included as control. A known mass of pre-oxidized SRB was added to 0.20 L of 5 mM dithionite solution in 50 mM citrate buffer (pH 6.4 ± 0.2). Reactors were shaken at 100 rpm and at different times 1-mL samples were withdrawn, filtered, and analyzed with DCPIP. Briefly, 2 mL of 10 mM DCPIP was mixed with 1-mL dithionite sample, diluted to 10 mL, allowed to react for 1 h, and 20-fold diluted for DCPIP analysis. EAC was then calculated using Eq. (1).

##### EDC measurement using ferricyanide

**Fig. 3:**
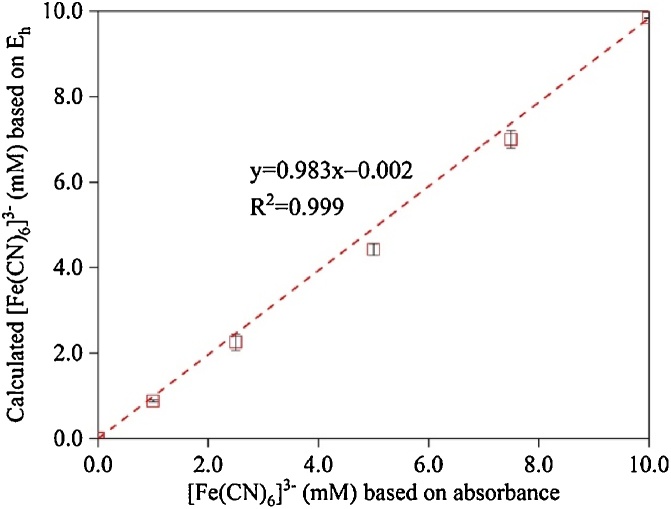
[Fe(III)(CN)_6_]^3−^ concentrations calculated using measured potentials (E_h_) and Nernst equation (Eq. (2)) vs. that obtained from absorbance at 420 nm. Error bars represent one standard deviation.

Each EDC measurement using ferricyanide was performed in duplicates, and reactors without SRB were included as control. The EDC of reduced SRB was measured in 0.25 L of 10 mM ferricyanide in 20 mM phosphate buffer (pH 7.0 ± 0.4). Reactors were shaken at 100 rpm, and 2.5-mL samples were taken at different times, filtered, and 10-fold diluted for ferricyanide measurement using a UV–vis spectrophotometer. Alternatively, the concentration of ferricyanide could be calculated using Eq. (2) based on the reduction potential (E_h_) of ferricyanide, which is pH-independent and can be measured using an ORP electrode. The ferricyanide concentrations based on absorbance and calculated from E_h_ and Nernst equation were almost identical (Fig. 3). With each ferricyanide concentration measured, the amount of electrons transferred from reduced SRB to Fe(III) was calculated using Eq. (1).

(2) $${\left[\text{F}\text{e}(\text{I}\text{I}\text{I}){\left(\text{C}\text{N}\right)}_{6}\right]}^{3-}\;(mM)\;=\frac{10^{\left[1+\frac{\left(E_{h}-0.43\right)}{0.059}\right]}}{10^{\left[\frac{\left(E_{h}-0.43\right)}{0.059}\right]}+1}$$

where E_h_ is the reduction potential (vs. standard hydrogen electrode) of ferricyanide solution measured using an ORP electrode.

#### ESC reversibility tests

Reversibility of ESC was evaluated over repeated cycles using Ti(III) citrate or dithionite as a reductant and ferricyanide or DO as an oxidant. The methods for EAC and EDC measurements were the same as those described above. All samples were processed in an anaerobic glove box except when they were to be air-oxidized next. SRB oxidation by DO was performed in 0.20 L continuously aerated DI water for 72 h. After each oxidation or reduction step, SRB sample was collected on a glass microfiber filter, rinsed thoroughly with (deoxygenated) DI water, vacuum-dried, and transferred to a desiccator for 24 h. Samples were weighed and divided into triplicates for the next EAC or EDC analysis. SRB mass loss (<10% between consecutive measurements) was accounted for in all EAC/EDC calculations, and all ESC values (in mmol e^−^/g SRB) are based on dry weight.
