# Supplementary material for: Chemical methods for determining the electron storage capacity of black carbon
Source: MethodsX. 2018 Nov 17;5:1515–20. doi: 10.1016/j.mex.2018.11.007 (PMC6260285; doi:10.1016/j.mex.2018.11.007)
Supplement: Supplementary file 1 [file mmc1.docx]

**Chemical Methods for Determining the Electron Storage Capacity of Black Carbon**

*Danhui Xin^1^, Minghan Xian^1,2^, and Pei C. Chiu^1*^*

^1^Department of Civil and Environmental Engineering, University of Delaware, Newark, DE 19716

^2^Department of Chemical and Biomolecular Engineering, University of Delaware, Newark, DE 19716

**Summary (3 pages; 4 figures)**

This Appendix contains 4 figures illustrating Ti(III) standardization and Ti(III) citrate, DCPIP, and ferricyanide calibrations.


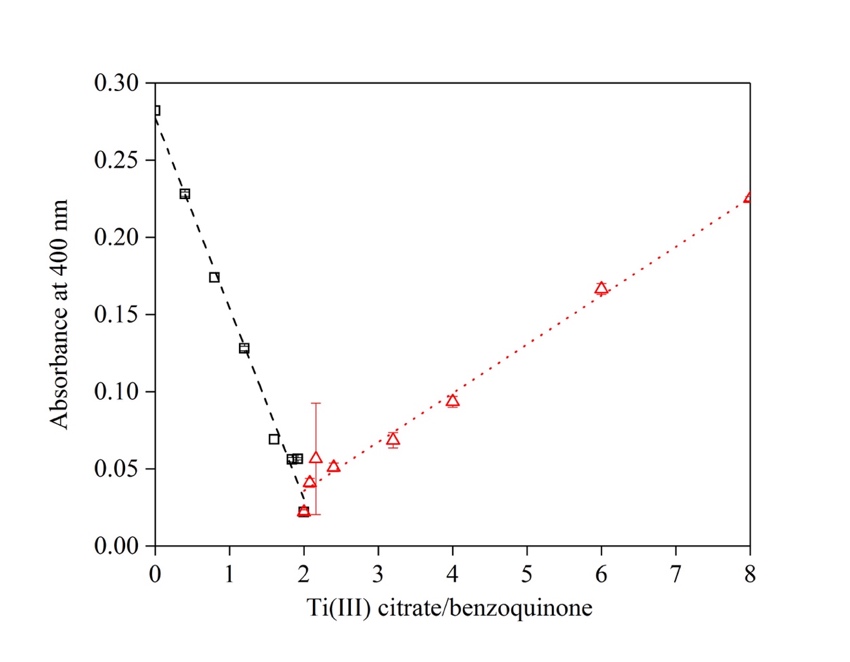


Figure S1. Calibration of Ti(III) in 100 mM citrate buffer using 1,4-benzoquinone at pH 6.4. Note that the two regression lines intercept at approximately 2, the stoichiometric ratio, where the solution absorbance was at a minimum, and the Ti(III) concentration could be determined accordingly. Error bars represent one standard deviation.

**
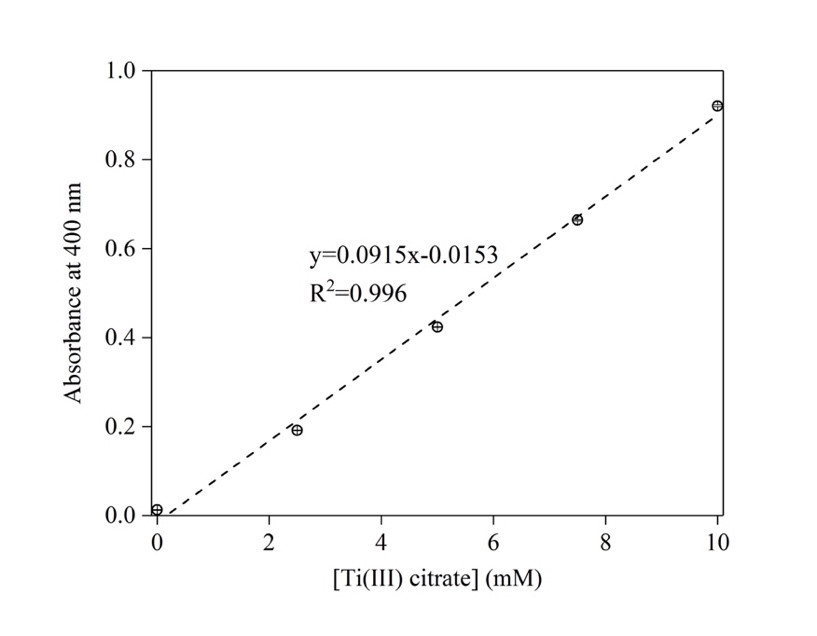
**

Figure S2. Calibration curve for Ti(III) citrate in 100 mM citrate buffer at pH 6.4. Error bars represent one standard deviation.

**
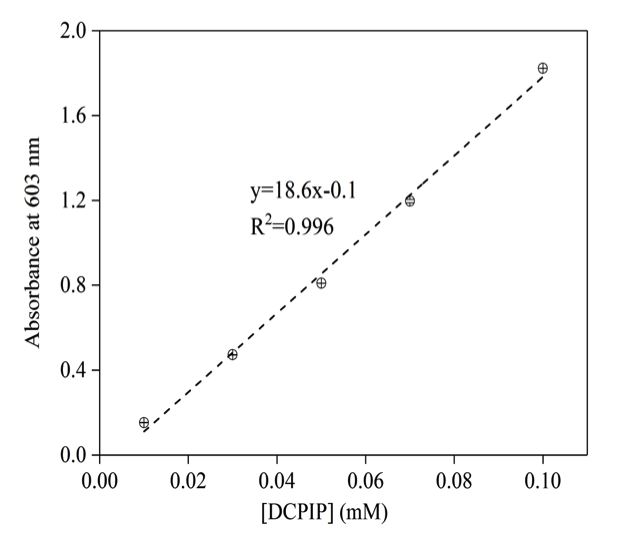
**

Figure S3. Calibration curve for DCPIP. Error bars represent one standard deviation.

**
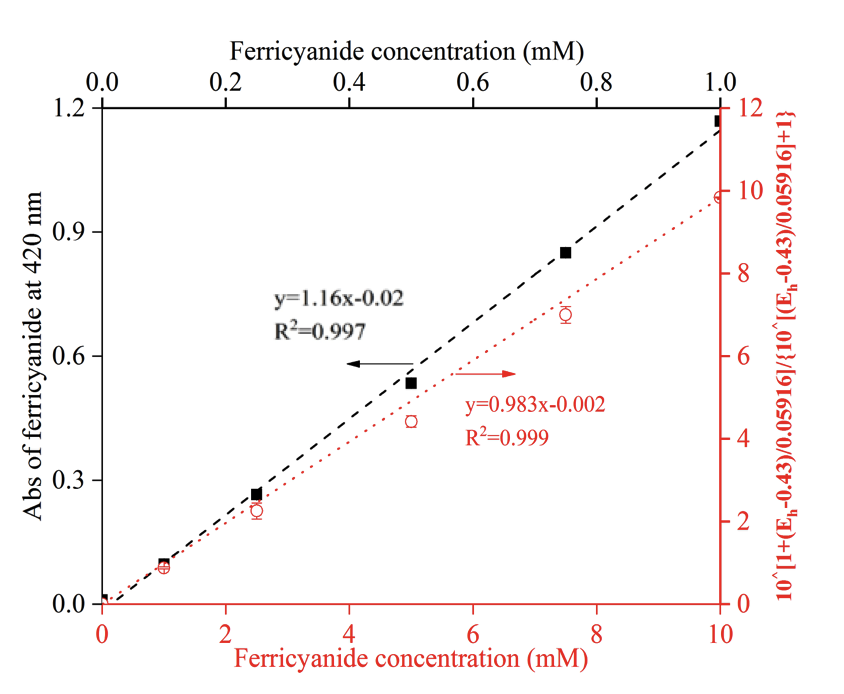
**

Figure S4. Calibration curve for ferricyanide in 20 mM phosphate buffer at pH 7.0. Error bars represent one standard deviation.
